# Stress level and associated factors among nurses working in the critical care unit and emergency rooms at comprehensive specialized hospitals in Southern Ethiopia, 2023: explanatory sequential mixed-method study

**DOI:** 10.1186/s12912-024-02004-w

**Published:** 2024-05-21

**Authors:** Getachew Nigussie Bolado, Bizuayehu Atinafu Ataro, Christian Kebede Gadabo, Agumas Shibabaw Ayana, Tamirat Ersino Kebamo, Worku Mimani Minuta

**Affiliations:** 1 Adult Health Nursing, School of Nursing, College of Health Science and Medicine, Wolaita Sodo University, Sodo, Ethiopia; 2 Pediatrics and Child Health Nursing, School of Nursing, College of Health Science and Medicine, Wolaita Sodo University, Sodo, Ethiopia; 3 Department of Anatomy, School of Nursing, College of Health Science and Medicine, Wolaita Sodo University, Sodo, Ethiopia; 4 Department of Medical Laboratory, College of Health Science and Medicine, Wolaita Sodo University, Sodo, Ethiopia; 5 Department of Public Health, Jinka University, Jinka, Ethiopia

**Keywords:** Emergency rooms, Ethiopia, Intensive care unit, Nurses, Stress level

## Abstract

**Background:**

Stress is a pervasive occurrence within certain professions, including nurses working in emergency and intensive care unit environments. Nurses in these settings often confront various stress-inducing factors, such as unsupportive management and distressing events like patient mortality, and experience notably higher levels of stress. Nevertheless, information is scarce regarding the precise level of stress in Ethiopia, particularly within southern hospitals.

**Objective:**

To assess stress levels and associated factors among nurses working in the critical care unit and emergency rooms at comprehensive specialized hospitals in southern Ethiopia, 2023.

**Methods:**

A facility-based cross-sectional explanatory sequential mixed-method study was undertaken, involving a total of 239 nurses. For the quantitative component, all nurses working in intensive care units and emergency rooms were included as participants, while a purposive sampling technique was employed to select participants for the qualitative aspect. Data for the quantitative study were gathered through the utilization of self-administered questionnaires, while interviews were conducted using a structured interview guide for the qualitative portion. Quantitative data entry and analysis were performed using EpiDataV4.6 and the Statistical Package for the Social Sciences software, respectively. Thematic analysis of the qualitative data was conducted using the OpenCode software.

**Results:**

The level of stress among nurses in the emergency and intensive care units was low (19.3%), moderate (55.9%), and high (24.8%). Workload (Adjusted odds ratio (AOR) = 3.51, 95% confidence interval (CI) (1.17–10.56) and time constraints (AOR = 2.5, 95% CI (1.03–6.07) were significantly associated with moderate stress level, while duty demands (AOR = 3.03, 95% CI (1.17–7.14), availability of medical equipment and supplies (AOR = 1.42, 95% CI (1.18–4.97), and witnessing death and dying (AOR = 2.34, 95% CI (1.13–5.88) were significantly associated with high-stress level. The qualitative data analysis revealed that the participants underscored the significant impact of organizational factors, individual factors, and profession-related factors on the stress levels experienced by nurses in emergency and critical care settings.

**Conclusion and recommendation:**

Based on the findings, the participants in this study experienced some level of stress, to varying degrees. Therefore, it is crucial to implement effective strategies such as optimizing staffing and workflow, improving communication and collaboration, providing adequate support and resources, leveraging technology and innovation, emphasizing patient-centered care, and implementing data-driven quality improvement to alleviate the burden.

## Introduction

Stress can be defined as a state of discomfort experienced by an individual, resulting from activities that are perceived as excessively intense and frequent. Such activities surpass an individual’s coping capabilities and available resources for effective management [1]. Another way to think of stress is as the body’s reaction to demands, either internal or external [2]. Stress stands as a pivotal phenomenon of our modern era, impacting multiple facets of our lives. It is pertinent to note that prolonged or high-stress levels can significantly influence various aspects of our existence [3]. High levels of stress are common in emergency and critical care unit (ICU) work, and if they are not well controlled, this can lead to decreased productivity and degraded quality of treatment [4].

The healthcare profession inherently entails significant stress, encompassing both routine stressors and critical incident events that have the potential to overwhelm an individual’s usual coping mechanisms. Nurses, who constitute a substantial portion of the healthcare workforce, are particularly susceptible to stressful conditions while delivering patient care [5]. It is anticipated that in the years to come, nurses will continue to endure higher levels of work-related stress, which directly impacts nurse and patient safety [6, 7]. Healthcare team members, particularly nurses, face numerous hazards at work and thus endure higher stress levels [8]. A study revealed that nurses providing treatment for patients with the severe acute respiratory syndrome (SARS) experienced significant levels of psychological distress. Recent studies indicate a marked increase in stress among nurses since the onset of the COVID-19 pandemic [9, 10].

The stress levels experienced by nurses working in emergency and critical care units are notably elevated [11]. The impact of stress on nurses is well-documented and can result in burnout, distress, and physical ailments. Prolonged exposure to stress can contribute to various health issues such as high blood pressure, fatigue, headaches, sleep disorders, and gastrointestinal problems. It is important to recognize that stress levels are influenced by three interconnected elements: individual factors, personal life circumstances, and the work environment. These factors collectively contribute to the level of stress experienced by nurses [12, 13]. Numerous global studies have shed light on stress prevalence levels among nurses. A study conducted in Pakistan showed that 7.4% experienced low stress, 80.2% moderate stress, and 12.4% high stress [14]. Another study in Kosovo revealed that 12.22%, 52.22%, and 35.55% of nurses experienced low, moderate, and higher stress respectively [15]. A study in Saudi Arabia showed 11.7% low stress, 87.8% moderate stress, and 1.3% high stress [1]. Further, a study in the United Arab Emirates revealed 14% low stress, 47% moderate stress, and 39% high stress levels among nurses [16].

Numerous factors contribute to the stress experienced by nurses working in Intensive Care Units (ICUs) and Emergency Rooms (ERs). Among these factors, organizational elements including high patient flow resulting in heavy workloads, suboptimal work environments, demanding duties, interpersonal challenges, and unsupportive managerial practices hold a prominent role. Likewise, personal factors such as individual personality traits, emotions, inadequate preparation or planning, familial and economic concerns, knowledge and skills, as well as the nature of dealing with death and dying, further contribute to stress levels. Nurses who face uncertainty about treatment outcomes and nurses who find themselves compelled to work during a pandemic due to financial constraints may endure heightened emotional and psychological stress. Sociodemographic factors may also play a significant role in influencing the heightened stress experienced by nurses in these settings [1, 3, 4, 7, 12, 17–21].

The repercussions of stress among nurses encompass a multitude of adverse outcomes. Turnover and work dissatisfaction are two of the most frequent effects of job stress. As a result, the company will have an exhausted unproductive staff [3]. Similarly, long-term, ongoing stress harms nurses’ health and can cause poor staff retention, organizational inefficiencies, and low job satisfaction [22]. Stress can manifest in physical effects on the body, resulting in fatigue, alterations in sex drive, gastrointestinal disturbances, and sleep disturbances. It can also have emotional ramifications, giving rise to anxiety, restlessness, diminished motivation or concentration, irritability, sadness, and even depression. Moreover, stress can potentially contribute to treatment errors, consequently jeopardizing the well-being and even the lives of patients [4, 23]. Extensive documentation exists regarding the impact of stress on the occurrence of preventable errors [2].

In Ethiopia, a developing nation with limited resources and inadequate health infrastructure, nurses assigned to intensive care units (ICUs) and emergency rooms (ERs) may confront substantial levels of stress. To the best of our knowledge, no previous research has been conducted in Ethiopia specifically focusing on nurses in these specific units. Hence, this study will be the inaugural endeavor of its kind in Ethiopia. While previous studies have explored the experiences of healthcare professionals as a whole, few have solely concentrated on nurses across various hospital wards. It is worth noting that the workload and contextual factors in ICUs and ERs differ significantly from those in other wards. Consequently, our primary objective is to evaluate the stress levels encountered by nurses in these critical units. This study also endeavors to not only determine the prevalence of stress but also assess the levels of stress, thereby facilitating the development of appropriate interventions categorized as low stress, moderate stress, and high stress. Furthermore, it is known that the majority of previous studies on this subject were solely conducted using a quantitative study design, which may not be sufficient for comprehensively identifying the factors associated with stress among nurses. Recognizing this limitation, the current study employed a mixed-method approach, specifically an explanatory sequential design, to enhance the depth and understanding of the research findings. Therefore, this study aimed to assess stress levels and associated factors among nurses working in the critical care units and emergency departments of selected comprehensive specialized hospitals in southern Ethiopia.

## Methods and materials

### Study area, design, and period

A cross-sectional mixed-method study was conducted at two specialized comprehensive hospitals in southern Ethiopia: Wachemo University Nigist Eleni Mohammed Memorial Comprehensive Specialized Hospital (WCUNEMMCSH), found in Hadiya Zone, and Wolaita Sodo University Comprehensive Specialized Hospital (WSUCSH), found in Wolaita Zone. These hospitals were specifically chosen because, in contrast to many primary and general hospitals in the region, they are comprehensive specialized hospitals owned by universities that provide a full range of patient care services and departments along with a substantial daily patient volume. The WCUNEMMCSH is located 230.9 km from Addis Ababa, serves around 1.5 million individuals, offers various inpatient departments and outpatient services, and has 263 nurses on staff, with 112 assigned to ICUs and ERs. The WSUCSH is situated about 300 km south of Addis Ababa, serves a population of 3.5–5 million, has 347 beds, and employs 291 nurses, including 127 in ICUs (main ICU, post-anesthesia care unit (PACU), neonatal intensive care unit (NICU), and ERs (adult emergency room (with surgical and medical emergency corners), pediatric emergency room, and maternal emergency room). The study took place from November 20 to December 10, 2023.

### Population

#### Source population

All nurses working in ICUs (main ICU, PACU, NICU) and ERs (adult ER (medical and surgical corners), pediatric ER, and maternal ER) in comprehensive specialized hospitals in Southern Ethiopia were considered as our source population for this study.

#### Study population

For the quantitative study, all staff nurses working in ICUs and ERs from both comprehensive specialized hospitals who were found during the data collection period and fulfilled the inclusion criteria were included, and for the qualitative study, all nurse leaders from both comprehensive specialized hospitals who were willing to participate in key informant interviews were included.

### Eligibility criteria

#### Inclusion criteria

In the quantitative study, nurses working in ICUs and ERs were eligible for inclusion if they possessed a minimum of six months of experience as full-time clinical nurses in the hospitals and had worked at least one month in ICUs and ERs. For the qualitative study, nurse leaders who met the following criteria were included: having at least six months’ experience as full-time clinical nurses in the hospitals, currently working in ICUs and ERs, and having a minimum of one month’s experience in these specific areas of practice.

#### Exclusion criteria

Nurses who were providing voluntary services in the hospitals because they serve for only a few months and are also not permanent employees, and those working in departments other than ICUs and ERs, were excluded from participation in this study.

### Sample size determination

The final sample size for the quantitative part of this study included all 239 nurses currently employed in ICUs and ERs within the two selected comprehensive and specialized hospitals. For the qualitative part of the study, the sample size was determined using a purposive sampling technique, specifically selecting leader nurses from ICUs and ERs. Participants were included in key informant interviews until content saturation was achieved, meaning that no new information or themes were emerging from the interviews.

### Study variables

#### Dependent variable

Stress level among nurses.

#### Independent variables

Sociodemographic characteristics (gender, age, marital status, educational status, work experience, working ward, monthly income, religion, ethnicity); organizational commitment factors (workload, working environment, duty demands, role demands, inter-personal relationships, unsupportive management, communication, medical supplies and equipment, work shift, job security, training opportunities, time constraints, task complexity); and personal factors (personality/emotion, individual plan, family issues, economic issues, technical knowledge and attitude, death and dying uncertainty concerning treatments, fear of infection).

### Data collection tools and procedures

In the quantitative study, data were collected through the utilization of pretested structured self-administered questionnaires. These questionnaires were obtained from previous studies conducted across different parts of the world [1, 4, 10, 18, 20, 22]. The questionnaire was originally prepared in English and subsequently translated into the Amharic language to check its consistency. To ensure consistency, the translated questionnaires were then back-translated into English. The questionnaires were organized into four distinct sub-sections. The first section of the questionnaire encompasses items about the sociodemographic characteristics of the study participants [1, 4, 10, 22]. The second part of the questionnaire focused on items to measure stress levels among nurses. In this study, the Perceived Stress Scale-10 (PSS-10) a 10-item self-report scale was used to examine the individual nurse’s stress levels obtained from previous study [1]. This tool contains 10 items and each item on the questionnaire was rated by nurses using a five-point scale, ranging from “never” (0) to “very often” [4]. The third and fourth sections of the questionnaire were specifically designed to assess the organizational and personal/individual factors that contribute to stress among nurses working in ICUs and ERs. These sections aimed to explore and understand the various aspects of the work environment and personal characteristics that influence the experience of stress in these healthcare settings.

### Operational definition and measurements

#### Level of stress

Stress can be described as the physiological, psychological, and emotional responses that individuals experience in response to the demands and challenges of life. Positive stress can serve as a motivator and propel individuals towards growth and achievement while negative stress occurs when these changes and needs overwhelm an individual, hindering their ability to cope and potentially leading to negative outcomes [3]. The respondents were requested to evaluate their feelings and thoughts over the previous month, and consequently, the total score for each participant can range from 0 to 40. The Perceived Stress Scale scores fall into the following categories: 0–13 for low stress, 14–26 for moderate stress, and 27–40 for severe stress. It is important to note that items 4, 5, 7, and 8 were scored in reverse manner. The PSS-10 questionnaire demonstrates good internal consistency, with a reliability coefficient (Cronbach’s alpha) of 0.78 [1].

### Data processing and analysis

Regarding the quantitative study, the collected data underwent a rigorous process of cleaning, coding, and entry into EpiData V4.6 software. Thereafter, the data were exported into Statistical Package for the Social Sciences (SPSS) Version 27 for analysis. Multinomial logistic regression analyses were performed to examine the relationship between the dependent and independent variables. Significant associations were obtained at an adjusted odds ratio (AOR) along with a 95% confidence interval (CI). For interpretation purposes, a *p*-value less than 0.05 was considered a statistically significant association. Finally, descriptive statistics, including tables, graphs, frequencies, and percentages, were utilized to depict the characteristics of the study sample and the responses obtained from the questionnaire items.

Regarding the qualitative study, the voice recordings conducted in the Amharic language were transcribed verbatim as soon as possible after the key informant interview, and detailed notes were also organized. Subsequently, the transcribed data were translated into English language and then imported into OpenCode 4.03 software for analysis. Thereafter, content thematic analysis was conducted to extract meaningful subthemes and themes from the data. Finally, original verbatim quotations from the participants were incorporated to support or enrich the findings obtained from the quantitative analysis. Then, the quantitative and qualitative data were combined and interpreted together in the discussion section to provide a more comprehensive understanding of the research question.

### Data quality control

In the quantitative study, a team consisting of six data collectors and two supervisors recruited from other health institutions received a day of training. Experts who were experienced in research reviewed the questionnaire and interview guide used in this study to ensure face validity. A pre-test was conducted on 10% of the sample size at Arba Minch General Hospital, which is not one of the target hospitals, a week before the actual data collection period. Based on the pre-test findings, necessary amendments, such as addressing unclear questions, typing errors, and ambiguous wording, were made. The internal consistency of the tool was assessed during the pre-test using Cronbach’s alpha (0.792), which demonstrated acceptable results for the target population. Before data entry and analysis, a manual check and cleaning process was undertaken.

For the qualitative study, a pre-tested interview guide which was prepared in English and then translated into Amharic language was used. Two researchers reviewed and compared the audio recordings with the transcribed notes to verify accuracy and completeness before translating the data.

Lincoln and Cuba’s trustworthiness criteria such as credibility, dependability, confirmability, and transferability, were ensured throughout this study. To ensure the credibility of the study, we established rapport and trust with the participating nurse leaders through prolonged engagement. We employed semi-structured interviews guided by interview questions and persistent observation to collect in-depth data. Peer debriefing with colleagues confirmed findings, while participant review ensured the accuracy of data representation. To enhance transferability, we clearly outlined the research design, data collection, and analysis processes. We also provided a thick description of the study context, including details that would allow readers to judge the transferability of the findings to other settings. To ensure dependability, after listening to the audio recordings of the interviews, both verbal and non-verbal data were recorded and the transcribed verbatim was saved properly to cross-check the whole process of the study and maintain the consistency of the interpretation. Confirmability was achieved by thoroughly documenting the research design, data analysis, and ethical considerations related to the interviews to enable readers to understand the methods and their effectiveness and to assess the trustworthiness of the findings.

## Results

### Sociodemographic characteristics of the study participants

Out of the total of 239 nurses who were assigned and are currently working in the Intensive Care Units (ICUs) and Emergency Rooms (ERs) of comprehensive specialized hospitals in southern Ethiopia, 238 willingly participated in this study, resulting in an impressive response rate of 99.6%. The mean age of the participating nurses was calculated to be 30.92, with a standard deviation of 6.3. Of the respondents, 136 (57.1%) were female, and more than half, 127 (53.4%), were followers of the Orthodox Christian faith. The majority of the participants, 101 (42.4%), were from Wolaita ethnic groups, and a substantial proportion, 155 (65.1%) of the respondents, reported being married. In terms of educational attainment, a substantial proportion of respondents, 144 (60.5%), held a bachelor’s degree. Similarly, a noteworthy number of individuals, 107 (45.0%), possessed 2–5 years of work experience, while an almost equal number, 105 participants (44.1%), had more than 5 years of work experience. The majority of the respondents, 155 (65.1%), reported that they earn a monthly income above 5295 Ethiopian Birr (ETB), and 133 (55.9%) of the participants were working in emergency rooms (Table 1).

**Table 1:** Sociodemographic characteristics of nurses working in the intensive care units and emergency rooms of comprehensive specialized hospitals in southern Ethiopia, 2023

Variable Name	Category	Frequency(*n*)	Percentage (%)
Age in years	20–29 years	101	42.4
	30–39 years	97	40.8
	*≥* 40 years	40	16.8
Gender	Female	136	57.1
	Male	102	42.9
Religion	Orthodox	127	53.4
	Protestant	98	41.2
	Muslim	11	4.6
	Others	2	0.8
Ethnicity	Wolaita	101	42.4
	Amhara	41	17.2
	Dawro	40	16.8
	Sidama	34	14.3
	Others	22	9.2
Marital status	Married	155	65.1
	Single	83	34.9
Educational status	Diploma	69	29.0
	First degree	144	60.5
	Second degree and above	25	10.5
Experience	< 2 years	26	10.9
	2–5 years	107	45.0
	> 5 years	105	44.1
Monthly salary	≤ 4085 ETB	26	10.9
	4086–5294 ETB	57	23.9
	≥ 5295 ETB	155	65.1
Ward	Intensive care units	105	44.1
	Emergency rooms	133	55.9

### Stress levels among nurses working in intensive care units and emergency rooms

**Fig. 1:**
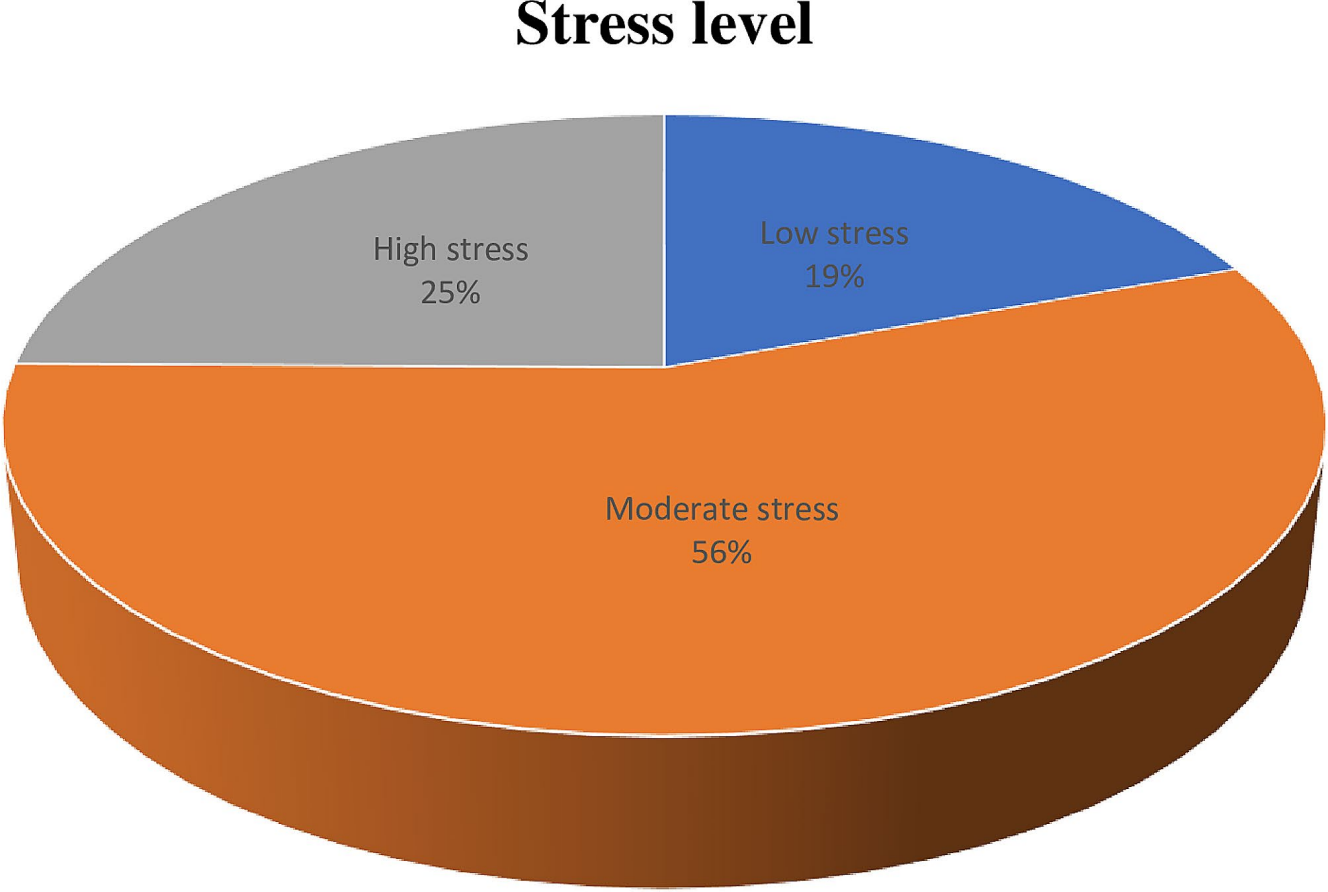
Stress level among nurses working in the critical care unit and emergency rooms at comprehensive specialized hospitals in southern Ethiopia, 2023

Among nurses working in the Intensive Care Units (ICUs) and Emergency Rooms (ERs) of comprehensive specialized hospitals in Southern Ethiopia, the prevalence of stress levels was as follows: low stress (19.3%), moderate stress (55.9%), and high stress (24.8%) (Fig. 1).

### Organizational and personal factors affecting the level of stress among nurses

#### Organizational factors

Among the study participants, 91 (38.2%) reported experiencing a medium workload, while 80 (33.6%) acknowledged having a high workload in the ICUs and ERs of the hospitals. Additionally, the majority of respondents, specifically 139 (58.4%), expressed satisfaction with their working environment, considering it to be favorable. Out of the respondents, a significant majority, 142 (59.7%), reported facing high duty demands, indicating a substantial level of obligations and responsibilities within their professional roles. Similarly, 117 (49.2%) acknowledged experiencing high role demands, denoting the elevated expectations and requirements associated with their specific positions or roles. Concerning interpersonal relationships, it was observed that a significant number of participants, 127 (53.4%), expressed having poor interpersonal relationships with both staff members and others within the hospital setting. On the other hand, a majority of respondents, 125 (52.5%), reported having good communication with others in the hospitals. A noteworthy proportion of the study participants, 144 (60.5%), expressed dissatisfaction with the supportiveness of the hospital management. Additionally, a significant majority of the respondents, 124 (52.1%), revealed that the medical equipment and supplies in the hospitals were insufficient, thereby placing additional stress on the nursing staff. Almost two-thirds of the study participants, 155 (65.1%), were working day shifts, and regarding job security, 145 (60.9%) of the respondents were unsatisfied with the job security in the ICUs and ERs of the hospitals. In terms of training opportunities, a significant proportion of the study participants, 145 (60.9%), reported that they hadn’t gotten training regarding the management of patients admitted to the ICUs and ERs in hospitals. Among the participants, a substantial majority, 128 (53.8%), reported having limited time constraints in their hospital duties. Furthermore, an equally considerable number of respondents, 132 (55.5%), described their tasks in hospitals as relatively simple (Table 2).

**Table 2:** Organizational factors affecting the level of stress among nurses working in the intensive care units and emergency rooms of comprehensive specialized hospitals in southern Ethiopia, 2023

Variable Name	Category	Frequency(*n*)	Percentage (%)
Workload	High	67	28.2
	Medium	91	38.2
	Low	80	33.6
Work environment	Unfavorable	99	41.6
	Favorable	139	58.4
Duty demands	High	142	59.7
	Low	96	40.3
Role demands	High	117	49.2
	Low	120	50.4
Interpersonal relationships	Poor	127	53.4
	Good	111	46.6
Management	Unsupportive	144	60.5
	Supportive	94	39.5
Communication	Poor	113	47.5
	Good	125	52.5
Medical equipment and supply	Inadequate	114	47.9
	Adequate	124	52.1
Duty shift	Night	83	34.9
	Day	155	65.1
Job security	No	145	60.9
	Yes	93	39.1
Training opportunity	No	142	59.7
	Yes	96	40.3
Time constraints	Limited	128	53.8
	Sufficient	110	46.2
Task complexity	Complex	106	44.5
	Simple	132	55.5

### Personal factors

Among the participants, the majority of individuals, 151 (63.4%), conveyed a positive disposition in terms of their emotions and personalities towards the situations they encountered. Regarding the individual plan or preparedness, 143 (60.1%) of the participants said that they had an adequate plan for their daily activities. Among the participants, a significant majority of individuals, 176 (73.9%), reported having no family issues, and in contrast, 139 respondents (58.4%) stated that they encountered economic issues or challenges. Out of the participants, 155 (65.1%) of them responded that they had adequate technical knowledge and skills for caring for patients. Death and dying proved to be a prevalent source of stress among nurses employed in the intensive care units (ICUs) and emergency rooms (ERs) of hospitals, as observed among the participants. A majority of individuals, 134 (56.3%), reported encountering such events, underscoring the significant emotional toll faced in these challenging contexts. A substantial proportion of the study participants, 167 (70.2%), identified the uncertainty of treatment as a particularly stressful situation. Additionally, 137 participants (57.6%) reported experiencing fear of infection while working in the intensive care unit and emergency rooms, highlighting the significant concerns and stresses associated with potential exposure to infectious diseases (Table 3).

**Table 3:** Personal factors affecting the level of stress among nurses working in the intensive care units and emergency rooms of comprehensive specialized hospitals in southern Ethiopia, 2023

Variable Name	Category	Frequency(*n*)	Percentage (%)
Personality	Negative	87	36.6
	Positive	151	63.4
Individual plan	Inadequate	95	39.9
	Adequate	143	60.1
Family issues	Yes	62	26.1
	No	176	73.9
Economic issues	Yes	139	58.4
	No	99	41.6
Technical knowledge and skills	Adequate	83	34.9
	Inadequate	155	65.1
Death and dying	Yes	134	56.3
	No	104	43.7
Uncertainty on treatment	Yes	71	29.8
	No	167	70.2
Fear of infection	Yes	137	57.6
	No	101	42.4

### Factors associated with high levels of stress among nurses

The outcome variable in this research was multinomial, denoting its classification into three distinct outcomes: low stress, moderate stress, and high stress. Consequently, multinomial logistic regression was employed for each independent variable, using the low-stress level as the reference category. Variables displaying a *p*-value of less than 0.05 were considered statistically significant factors influencing the level of stress among nurses working in intensive care units (ICUs) and emergency rooms (ERs). Findings indicate that the variables of workload, and time constraints were significantly associated with moderate stress levels, while duty demands, medical equipment, and supplies, as well as death and dying, were significantly associated with higher levels of stress (*p*-value < 0.05).

The odds of having moderate stress were 3.51 times higher in those nurses who had a high workload than those who had a low workload in ICUs and ERs (AOR = 3.51, 95% CI (1.17–10.56). Similarly, nurses who had limited time had 2.5 times the odds of having moderate stress than those who had sufficient time (AOR = 2.5, 95% CI (1.03–6.07). Those nurses who had high duty demands had 3.03 times the odds of having high stress than those who had low duty demands (AOR = 3.03, 95% CI (1.17–7.14)). The odds of having high stress were 1.42 times higher in nurses who lacked medical equipment and supplies in ICUs and ERs than in those who had it (AOR = 1.42, 95% CI (1.18–4.97)). The odds of having high stress were 2.34 times higher in nurses who witnessed death and dying at ICUs and ERs than in those who hadn’t experienced it (AOR = 2.34, 95% CI (1.13–5.88) (Table 4).

**Table 4:** Factors associated with the level of stress among nurses working in the intensive care units and emergency rooms of comprehensive specialized hospitals in southern Ethiopia, 2023

Variable	Associated with	*P*-value	Exp(B)	95% CI		Reference
				Lower	Higher	
Workload	Moderate stress	0.026	3.51	1.17	10.56	Low
Time constraints	Moderate stress	0.043	2.5	1.03	6.07	Sufficient
Duty demands	High stress	0.022	3.03	1.17	7.84	Low
Medical equipments and supplies	High stress	0.042	1.42	1.18	4.97	Adequate
Death and dying	High stress	0.026	2.34	1.13	5.88	No

### Result from key informant interview

During the key informant interviews, a total of seven nurse leaders from intensive care units and emergency rooms of comprehensive and specialized hospitals in southern Ethiopia were recruited and actively participated. The age range of the participants varied from 26 to 41 years old, encompassing individuals with educational qualifications ranging from a Bachelor of Science degree to holders of master’s degrees (Table 5). Through the process of data analysis, the utilization of OpenCode 4.02 software revealed the emergence of three main themes, along with eleven subthemes. The main theme categories were factors related to the organization, individual factors related to nurses, and factors associated with the nursing profession (Table 6).

**Table 5:** Socio-demographic characteristics of nurses who participated in key informant interviews at intensive care units and emergency rooms of comprehensive specialized hospitals in southern Ethiopia, 2023

Participant ID	Age in years	Sex	Academic Rank	Working ward
NL-01	33	Female	Bachelor degree	ICU
NL-02	26	Female	Bachelor degree	ICU
NL-03	29	Male	Bachelor degree	ER
NL-04	32	Female	Bachelor degree	ICU
NL-05	36	Female	Master’s degree	ICU
NL-06	30	Female	Bachelor degree	ER
NL-07	41	Male	Master’s degree	ER

*NL = Nurse Leader*

**Table 6:** Themes and sub-themes emerged from key informant interviews by using OpenCode 4.03 software for factors influencing stress levels among nurses working in the intensive care units and emergency rooms of comprehensive specialized hospitals in southern Ethiopia, 2023

Themes	Subthemes
Factors related to organization	• Lack of facilities and personal protective equipments • High workload in the ICUs and ERs • Lack of support from managers and leaders • Lack of training opportunities
Individual factors related to nurse	• Pressure from families • Poor communication skills
Factors related to nursing profession	• Job insecurity • Fear of infection transmission • Low salaries and benefits • Witnessing deaths and dying • Complexity of duties, high role expectations and responsibilities

### Theme one: factors related to the organization

During the key informant interviews, the participants underscored the significant impact of organizational factors on the stress levels experienced by nurses in emergency and critical care settings. They highlighted the detrimental effects of inadequate facilities and personal protective equipment, excessive workloads, insufficient support from managers and leaders, and limited training opportunities. These organizational challenges were identified as key contributors to the heightened stress experienced by nurses in these demanding environments.

#### Subtheme 1: lack of facilities and personal protective equipment

*“…Within the intensive care unit, a poignant issue arises as the availability of essential protective gear such as gloves, gowns, goggles, face masks, and disinfecting alcohol falls significantly short. This intricate predicament fuels a profound sense of dissatisfaction concerning my work within this unit. The primary concern revolves around the constant fear of potential transmission of infections between patients and healthcare professionals, thereby compromising both our well-being and that of the patients we serve.”***(NL-01)**.

*“…I frequently find myself in circumstances where the essential infrastructure and personal protective equipment are lacking. It’s concerning because I feel vulnerable and unable to properly care for my patients. It adds a layer of stress and anxiety to an already demanding environment.”* (**NL-02**).

*“…The scarcity of resources impedes the attainment of optimal satisfaction levels among nurses working within intensive care units (ICUs) and emergency rooms (ERs) within our hospital. The allocation of resources, amid wards burdened with high patient flow and demanding workloads, falls below the necessary threshold. Thus, all relevant stakeholders within the hospital must take diligent notice of this matter.***(NL-05)**.

*“…The inadequacy of essential facilities and infrastructure stands as a paramount stress-inducing factor for nurses working in intensive care units (ICUs) and emergency rooms (ERs) while delivering patient care. The presence of vital resources, equipment, and cutting-edge technology constitutes integral elements within ICUs and ERs. Regrettably, our hospital frequently suffers from insufficiencies in the provision of necessary medical supplies and equipment, impeding the smooth facilitation of patient care within these settings.”***(NL-07)**.

#### Subtheme 2: high workload in the ICUs and ERs

*“…It feels like an endless battle inside. The ERs are overflowing with patients at all times. The nurses are running all over the place, hardly having time to breathe. Their faces convey the stress and urgency that they are feeling. Not only is it overwhelming us, but it’s also overwhelming them. I have no idea how they handle everything.“***(NL-03)**.

*“…As we all know, the designated domains encompassed by intensive care units and emergency rooms within hospital settings are inherently characterized by their intense nature. A consequential expectation of these environments involves the perpetually elevated workload imposed upon us, profoundly influencing our productivity and precipitating elevated levels of stress for us. Thus, this observation underscores the perpetual bustling atmosphere and ensuing strain borne by nurses as they contend with the demanding toll wrought by workload-induced stress within ICUs and ERs.”* **(NL-06).**

*“…Working in emergency rooms presents constant challenges for nurses due to the high-stress nature of the healthcare environments there.”***(NL-07)**.

#### Subtheme 3: lack of support from hospital managers and leaders

*“…It might be disappointing when hospital administrators and executives don’t offer the required assistance financially or at least morally. As nurses, we frequently find ourselves stumbling through difficult circumstances without the support or direction we require. It adds to the already high levels of stress we encounter in emergency rooms and critical care units by leaving us feeling helpless and overwhelmed.“***(NL-05)**.

*“…In addition, a noteworthy concern revolves around the absence of recognition and motivation from hospital leaders extended to nurses toiling within the demanding confines of emergency and intensive care units. The dearth of such essential factors has the potential to diminish their vigor, exacerbate stress levels, and impede their ability to perform optimally, falling short of the expected standard.”***(NL-06)**.

#### Subtheme 4: lack of training opportunities

*“…I’m not aware of any training programs for nurses who work in intensive care units or emergency rooms. It’s possible to say there are not many training options, but none exist at all, especially ones that focus on intensive care units and emergency rooms. Professionals need the training to handle stressful events in high-stress settings, like fatalities, and also enhance nurses’ knowledge and attitudes to deliver high-quality patient care.”***(NL-04)**.

### Theme two: individual factors related to nurses

The data obtained from key informant interviews conducted by nurse leaders shed light on the individual factors affecting nurses working in emergency and intensive care settings, such as family pressures and poor communication skills. These findings emphasize the individual challenges that significantly contribute to the increased stress levels experienced by nurses in these roles.

#### Subtheme 1: pressure from family

*“…There are instances when the weight of familial pressure feels intolerable. Their persistent desire for me to quit this profession stems from their limited understanding and low expectations regarding the benefits and rewards it offers. I would find immense happiness if my family could acknowledge the unique demands of my chosen path and offer unwavering support instead of exacerbating the existing stress.”****(NL-01)***.

*“…My family, particularly my father, and sister, persistently implored me to resign from my position, subjecting me to psychological stress. Despite my best efforts to persuade them otherwise, their resistance remains steadfast. Their reasoning stems from the notion that the remuneration derived from my chosen profession pales in comparison to their monthly earnings as traders. Thus, they ardently advocate for me to venture into alternative business pursuits, casting doubt on the viability of sustaining my current professional trajectory.”***(NL-06)**.

#### Subtheme 2: poor communication skills

*“…. Sometimes, the tension between us affects the whole team because of poor communication skills. It’s hard to find support when the atmosphere is stressful, and it is also frustrating when clear communication could make such a difference. I want to trust that my concerns are understood, but it’s not always the case.“***(NL-04)**.

*“…I’d love to have a deeper sense of togetherness among nurses working the stressful settings like ERs and ICUs and have good communication skills. Stress among nurses in the ward or unit can be caused by a lack of trust, cooperation, and effective communication, which can have a very negative impact on us. Although we’re meant to work as a team, there are moments when it seems like we’re alone.“***(NL-03)**.

*“…Uncertainty brought on by a breakdown in communication can be distressing. Not feeling heard can be difficult, particularly when it pertains to your health. It intensifies an already stressful atmosphere by adding another layer of tension.“***(NL-05)**.

### Theme three: factors associated with the nursing profession

This theme underscores the rigorous and ever-evolving nature of nursing in critical care settings, shining a spotlight on the distinct challenges and stressors that are inherent to the nursing profession in emergency and ICU environments. These include concerns related to job security, the pervasive fear of infection transmission, the impact of low salaries and inadequate benefits, the emotional weight of witnessing deaths and experiencing the dying process, the intricate complexities of daily duties, and the weighty expectations and responsibilities placed upon nurses in these settings.

#### Subtheme 1: job insecurity

*“…As nurses laboring within these demanding and intense environments, we encounter multifarious challenges, and among them is the pervasive specter of job insecurity. Regrettably, we occasionally find ourselves subjected to physical aggression from patients or exposed to threats emanating from their attendants. These distressing circumstances engender a palpable atmosphere of apprehension within the confines of ICUs and ERs, exacerbating the existing stressors in our professional domain.”***(NL-02)**.

*“…I’ve noticed the turnover among the nurses due to threats from patients and lack of job security in general. Due to this, it makes me wonder about the consistency of care. If there’s always someone new, it’s tough to build that trust and familiarity you need when you’re in a vulnerable state.“***(NL-06)**.

#### Subtheme 2: fear of infection transmission

*“…The fear of contracting infections from patients stands as one of the foremost sources of stress for nurses operating within ICUs and ERs. Particularly during pandemics such as the Corona outbreak, this unease is greatly intensified. The sight of nurses meticulously donning their protective gear serves as a stark reminder of the imminent threat of contagion. One cannot help but empathize with their palpable apprehension, as it becomes inexorably intertwined with our own shared concerns. Though one strives to maintain faith in their safety, the ever-present nature of this worry persists, amplified by the knowledge of the high-risk milieu in which we find ourselves.”***(NL-07)**.

#### Subtheme 3: low salaries and benefits

*“…It is frustrating when we put our heart and soul into this job, but still we struggle to meet our basic needs. We work long and challenging hours, and it feels disheartening to see that the demands of the job don’t match the salaries we get. This makes us feel undervalued and takes a toll on our morale and overall job satisfaction.”***(NL-01)**.

*“…One of the main sources of stress is the financial burden. While we are here to help patients through some of their most trying times, it can be difficult when we are also worrying about money. It’s a constant burden that can negatively affect our mental and emotional health as well as how we approach our work.“***(NL-06)**.

*“…We’re dedicated to this profession, but it’s disappointing to see our hard work and commitment undervalued in our salary. It’s hard not to bring that home with you, knowing that you’re sacrificing so much and yet struggling financially. The stress of feeling inadequately compensated impacts our ability to give our best at work.“***(NL-07)**.

#### Subtheme 4: witnessing deaths and dying

*“…As nurses working in emergency departments and critical care units, we frequently see the heartbreaking reality of deaths and dying. Being directly involved in such a significant loss has a tremendous emotional cost. Every loss serves as a sobering reminder of our frailty and limitations, which raises stress and emotional discomfort levels.“***(NL-01)**.

*“…Nurses may experience severe distress when they witness death and dying in emergency and intensive care units. Seeing patients and their loved ones’ suffering and sorrow while knowing that our efforts might not always result in a favorable conclusion can be stressful. These events have a profound impact on our emotions and minds, which raises stress levels and may have long-term emotional repercussions.“***(NL-02)**.

*“…One of the most difficult parts of our work is being in the front of situations involving life and death. Although we’re educated to deal with it, it’s never simple. It’s hard not to feel the weight of such experiences when you see the pain and loss every day. It undoubtedly increases the emotional load, which eventually causes tension and emotional tiredness.“***(NL-05)**.

*“…It’s extremely difficult to balance giving high-quality care with managing the emotional impacts of dying and recurrent deaths. Although you want to support your patients and their families, the cumulative impact of these encounters can be rather stressful. Maintaining mental resilience in the face of the realities of our workplace is a never-ending battle.“***(NL-04)**.

*It can be heartbreaking how frequently we see people in the emergency departments and critical care units passing away. Nurses experience high levels of stress as a result of their ongoing exposure to life’s unpredictable fragile nature, which detrimentally impacts their emotional health and general capacity to handle work-related obligations.“***(NL-07)**.

#### Subtheme 5: the complexity of duties, high role expectations, and responsibilities

*“…Nurses have a heavy burden due to the complexity of our work in the emergency and intensive care units. We manage a broad range of duties, such as monitoring vital signs, administering medications, arranging care with different medical specialists, and continuously adjusting to circumstances that are changing quickly. Due to the demanding nature of these duties, we may experience higher levels of stress as we work under pressure to remain productive and provide the best service possible.“***(NL-05)**.

*“…We (nurses) working in emergency departments and critical care units face tremendous pressure and higher levels of stress due to the high role expectations placed upon us. When providing treatment to critically ill patients, we are expected to be extremely competent, informed, and effective.”***(NL-04)**.

*“…Our responsibilities include giving our patients the best treatment possible, standing up for them, collaborating with medical teams efficiently, and making important choices under pressure.“***(NL-07)**.

## Discussion

This study aimed to determine the level of stress experienced by nurses working in emergency rooms and intensive care units within comprehensive and specialized hospitals in southern Ethiopia. Additionally, the study aimed to identify the factors that were associated with the levels of stress among these nurses. This study revealed that the low, moderate, and high stress levels were 19.3%, 55.9%, and 24.8%, respectively. This finding was higher than the study conducted in Saudi Arabia [1] and Kosovo [15]. This difference might be due to differences in study time gaps, study areas, and study participants (for instance, the study participants of the above study were nurses from all settings, but our study focused on nurses from intensive care units and emergency departments). The other possible reasons for this difference might include high patient-to-nurse ratios, a shortage of essential medical supplies, inadequate infrastructure, a lack of training opportunities, limited access to technology and advanced medical equipment, as well as limited access to support systems such as counseling, mental health resources, and debriefing mechanisms following stressful or traumatic patient events as compared to developed nations. Similarly, this difference might be because, beyond the specific healthcare environment, broader systemic and societal factors can contribute to higher stress levels among nurses in our study area and Ethiopia at large. Economic constraints and societal stressors may compound the emotional and mental burden nurses face.

However, our finding was lower than the study conducted in Pakistan [14] and United Arab [16]. This difference might be due to the difference in sociodemographic characteristics, and study time gaps and as well there might be a stronger sense of community and support among nurses and other healthcare professionals in our study area, providing a network of emotional and professional support that mitigates stress levels. This communal support can help in sharing the burden of high-stress situations. An alternative explanation for the disparity could be attributed to the high adaptability displayed by nurses in Ethiopia, attributable to their experience of working in resource-limited settings. This adaptability and resilience might contribute to the development of distinctive stress management and coping strategies among nurses. Furthermore, the presence of strong familial and cultural support systems in Ethiopia may also provide additional emotional and psychological support to nurses, potentially leading to lower levels of stress compared to other contexts.

The workload was the first significant factor associated with moderate stress. The odds of having moderate stress were 3.51 times higher in those nurses who had a high workload than those who had a low workload in ICUs and ERs. This finding was in line with the study findings conducted in Pakistan [14], Texas [2], Rwanda [24], and East Gojjam [6]. This could be because nurses are expected to care for more critically sick patients with complicated needs while they have a busy workload, which frequently reflects increased demands and obligations. Prolonged periods of intense activity, fewer rest intervals, and increased degrees of both physical and mental stress may be the cause. In addition, a heavy workload may prevent nurses from taking advantage of necessary leisure to rest and rejuvenate and from engaging in self-care, pleasure, and introspection. This may make nurses feel even more stressed, which would be extremely detrimental to their general well-being.

This finding is supported by the qualitative data, in which nurses described how excessive workload, which was coded under the theme of ‘factors related to the organization’, can lead to feelings of overwhelm, exhaustion, and burnout. More than half of key informant interview participants emphasized that the considerable workload imposed on them in emergency rooms and intensive care units significantly impacts their productivity, resulting in increased levels of stress. This burden not only affects their professional lives but also has negative effects on their social well-being and can lead to physical exhaustion, further compounding the challenges faced by these nurses.

Similarly, time constraints had a significant association with nurses’ moderate stress. Those nurses who had limited time had 2.5 times the odds of having moderate stress than those who had sufficient time. This finding was congruent with the study conducted in Pakistan [25] and Saudi Arabia [4]. The possible reason for this might be due to time constraints, increased job demands, and the difficulties of managing critical care situations within short timeframes, which can result in moderate to higher levels of stress for nurses who have limited time. On the other hand, nurses who possess ample time may be able to better handle their professional obligations, which in turn lowers stress levels.

This finding was supported by the data from key informant interviews in the qualitative component of the study, which was coded under the theme of ‘factors related to the organization’, which indicated that nurses who have limited time may experience pressure to balance several important responsibilities, which can increase their sense of urgency and make it more difficult for them to successfully manage their workload. Because of these conditions, nurses may experience moderate to higher levels of stress as they work to provide high-quality care while under time pressure, which can result in feelings of pressure, anxiety, and emotional strain.

There were also factors associated with high stress among nurses working in emergency rooms and intensive care units. Nurses’ duty demand was one of the factors significantly associated with high stress levels among nurses. Those nurses who had high duty demands had 3.03 times the odds of having high stress than those who had low duty demands. This finding was similar to the study conducted in Pakistan [25]. This might be due to the increased workload and the emotional, cognitive, and physical demands of managing a higher volume of tasks, nurses who are faced with high-duty demands are more likely to have increased stress levels. Managing a large number of patients, dealing with complicated medical issues, and resolving critical care circumstances when resources are limited are some examples of high-duty demands.

This finding was also supported by the qualitative part of the study, in which nurses described how increased duty demand, which was coded under the theme of ‘factors related to the organization’, can lead to high stress among nurses. They expressed that having high duty demands due to the complexity of their work in the emergency and intensive care units, such as monitoring vital signs, administering medications, arranging care with different medical specialists, and continuously adjusting to circumstances that are changing quickly, may cause them to experience higher levels of stress as they work under pressure to remain productive and provide the best service possible.

Likewise, the lack of medical equipment and supplies in ERs and ICUs was another factor significantly affecting high levels of stress among nurses. The odds of having high stress were 1.42 times higher in nurses who lacked medical equipment and supplies in ICUs and ERs than in those who had it. This was congruent with the findings of two studies conducted in Pakistan [14, 25]. The possible reason for this might be the fact that when nurses in ICUs and ERs do not have enough medical equipment and supplies, they experience more stress because it is hard to provide care when there are not enough resources, such as personal protective equipment. On the other hand, when nurses have the equipment and supplies they need, they experience less stress because they can take better care of patients and create a safer and more efficient environment.

This was supported by a qualitative finding coded under the theme of ‘factors related to the organization’, in which the majority of the participants explained that the insufficient availability of crucial protective equipment, including gloves, gowns, goggles, face masks, alcohol, etc., leads to a pronounced dissatisfaction with my work in this unit. This scarcity not only compromises nurses’ well-being but also jeopardizes the safety and health of patients. Additionally, it exposes nurses to a heightened risk of contagious infections, further exacerbating the stress and anxiety already prevailing in this challenging environment.

Finally, witnessing death and dying was significantly associated with high-stress levels among nurses working in ERs and ICUs. The odds of having high stress were 2.34 times higher in nurses who witnessed death and dying at ICUs and ERs than in those who hadn’t experienced it. The studies conducted in Pakistan [14], Rwanda [24], and East Gojjam [6] had similar findings with this. This might be because seeing death and dying in intensive care units and emergency rooms can have a profound emotional and psychological impact on nurses. As they manage the challenges of providing care in these emotionally stressful situations, they may experience compassion fatigue, emotional exhaustion, and increased stress. On the other hand, nurses who have not encountered these circumstances could be less likely to go through such a major emotional and psychological strain, which would lead to relatively lower stress levels.

This finding is supported by the qualitative data coded under the theme of ‘individual factors related to nurses’, which indicated that stress levels were extremely high for nurses working in emergency and intensive care units when they saw patients die. They clarified that it can be upsetting to witness patients’ and their loved ones’ anguish and suffering while understanding that their efforts might not always lead to a successful outcome.

## Implications of the study

For nursing theory, the findings of this study can contribute to the formulation of new conceptual frameworks that integrate the multifaceted factors influencing stress levels, thereby enhancing the understanding of stress within nursing theory. For nursing practice, the findings can help identify the factors influencing stress levels among critical care and emergency nurses and offer valuable insights into the specific challenges and stressors faced in these environments. This knowledge can inform the development of tailored interventions, stress management protocols, and support systems designed to address the unique stressors encountered by nurses in these settings. For nursing research, the finding might serve as a foundation for further research endeavors aimed at exploring and addressing stress among nurses in critical care and emergency units. It opens the door for continued investigation into specific stress mitigation strategies, longitudinal studies assessing the efficacy of interventions, and exploration of additional factors influencing stress in these environments.

## Conclusion and recommendations

This study aimed to assess the stress levels experienced by nurses working in emergency rooms and intensive care units at comprehensive specialized hospitals in southern Ethiopia. The findings revealed that both moderate and high levels of stress among nurses were considered high. Workload and time constraints were significantly associated with moderate stress levels, whereas duty demands, availability of medical equipment and supplies, and witnessing death and dying were significantly associated with high stress levels.

Based on these findings, it is strongly recommended that comprehensive specialized hospitals and the Ethiopian government, particularly the Ministry of Health, take proactive measures to mitigate stress and its contributing factors among nurses by implementing appropriate strategies. These strategies should be designed to address the specific challenges faced by nurses in emergency rooms and intensive care units. Essential recommendations include ensuring the provision of necessary facilities, such as personal protective equipment, organizing diverse training opportunities, enhancing benefits and salaries to enhance nurses’ job satisfaction, and implementing regular supervision and support systems for nurses. Future researchers are recommended to conduct an observational study to investigate the stress levels among nurses and the factors that contribute to it. This will allow for a thorough examination of the findings and assess their applicability to a broader population.

### Strengths and limitations of the study

The strength of the study was that it incorporated all nurses working in both comprehensive specialized hospitals in southern Ethiopia. The other strength was the use of an explanatory sequential mixed study design to further enhance the quantitative findings, adding depth and context to the research. However, certain limitations should be acknowledged. Firstly, the cross-sectional nature of the study design restricts the ability to establish a causal relationship between the dependent and independent variables. Secondly, the reliance on self-administered questionnaires for data collection introduces the potential for response bias among participants.

## Data Availability

The datasets used and/or analyzed during the current study are available from the corresponding author upon reasonable request.

## Abbreviations

AOR: Adjusted Odds Ratio
CI: Confidence Interval
COVID-19: Coronavirus Disease 2019
ER: Emergency Room
ICU: Intensive Care Unit
NICU: Neonatal Intensive Care Unit
PACU: Post-anesthesia Care Unit
SARS: Severe Acute Respiratory Syndrome
SPSS: Statistical Package for the Social Sciences
WCUNEMMCSH: Wachemo University Nigist Eleni Mohammed Memorial Comprehensive Specialized Hospital
WSUCSH: Wolaita Sodo University Comprehensive Specialized Hospital
